# A comparative cross-sectional evaluation of generative AI chatbots for patient-oriented bacterial vaginosis health advice: safety, accuracy, guideline concordance, empathy, and readability

**DOI:** 10.3389/frph.2026.1967818

**Published:** 2026-09-17

**Authors:** Ling Miao, Lina Gu, Zhaole Gong, Zhengfeng Gu, Xiaoli Qian

**Affiliations:** 1 Department of Gynecology, Nanquan Branch, Xuelang Subdistrict Community Health Service Center, Wuxi, Jiangsu, China; 2 Department of Pain Medicine, The Affiliated Wuxi People's Hospital of Nanjing Medical University, Wuxi People's Hospital, Wuxi Medical Center, Nanjing Medical University, Wuxi, Jiangsu, China; 3 Department of Pediatrics, Mudanjiang Second People's Hospital, Mudanjiang, Heilongjiang, China; 4 Department of Interventional Radiology, The Affiliated Wuxi People's Hospital of Nanjing Medical University, Wuxi People's Hospital, Wuxi Medical Center, Nanjing Medical University, Wuxi, Jiangsu, China

**Keywords:** bacterial vaginosis, chatbot, generative artificial intelligence, large language model, patient education, patient safety

## Abstract

**Background:**

Bacterial vaginosis (BV) concerns involve intimate symptoms, stigma, diagnostic uncertainty, medication use, pregnancy, sexual health, and self-care. Publicly accessible generative artificial intelligence chatbots offer immediate and potentially non-judgmental information, but the quality and safety of specific responses remain uncertain.

**Objective:**

To compare five publicly accessible generative AI chatbot interfaces using standardized, researcher-developed patient-oriented questions about BV. In this study, patient-oriented denotes consumer-facing wording and does not imply direct patient derivation or validation.

**Methods:**

Sixty-one standardized English-language questions were submitted once to ChatGPT, Gemini, Microsoft Copilot, DeepSeek, and Doubao in separate single-turn sessions, yielding 305 responses. Five senior obstetrician-gynecologists independently evaluated safety, accuracy, study-specific guideline-anchored concordance, and empathy using a question-specific reference framework. Independent ratings were locked before adjudication and were used to estimate inter-rater agreement. Consensus scores were used for primary comparisons, and a sensitivity analysis used the median of the five locked ratings. Readability was assessed separately using six formula-based indices. Paired comparisons used Cochran's Q, Friedman, McNemar, and Wilcoxon signed-rank tests with Benjamini-Hochberg adjustment.

**Results:**

Thirty-one responses (10.2%) were classified as unsafe or potentially unsafe. Observed interface-specific rates ranged from 6.6% (95% CI, 2.6%–15.7%) to 14.8% (95% CI, 8.0%–25.7%), while the matched binary comparison did not detect an overall difference (Cochran's Q = 2.596, df = 4, *P* = 0.627). Under the documented query conditions, differences were detected in accuracy (Kendall's W = 0.686), study-specific guideline-anchored concordance (W = 0.243), empathy (W = 0.322), and all six readability indices (W range, 0.482–0.679; all *P* < 0.001). The sensitivity analysis produced the same inferential conclusions. Unsafe content occurred in every interface and was concentrated in recurrent-BV, sexual-health, medication, and self-care scenarios.

**Conclusions:**

This exploratory study characterizes a recorded sample of specific chatbot responses, not stable or repeatable performance characteristics. Clinically relevant risks were observed in every interface, and the non-significant safety comparison should not be interpreted as equivalence. Because each researcher-developed prompt was submitted once and the interfaces were queried on different dates in a fixed order, the findings do not establish a stable ranking of underlying model capability, clinical effectiveness, or suitability for unsupervised care.

## Introduction

Bacterial vaginosis (BV) is a common vaginal dysbiosis characterized by reduced protective Lactobacillus species, increased anaerobic diversity, and higher vaginal pH (1). Many affected individuals are asymptomatic; when symptoms occur, thin gray-white discharge and a fishy odor are typical. Prominent pruritus, vulvar erythema, green or yellow discharge, pelvic pain, fever, bleeding, or dyspareunia should not automatically be attributed to BV because candidiasis, trichomoniasis, cervicitis, pelvic inflammatory disease, a retained foreign body, and other conditions may present similarly (2).

BV is associated with sexual and microbiome-related factors but is not proof of infidelity, promiscuity, poor hygiene, or a classic sexually transmitted infection. Douching and intravaginal cleansing may worsen vaginal imbalance (3). Symptomatic BV may require oral or intravaginal antimicrobial treatment, with pregnancy, adverse effects, route preference, and local guidance affecting management (4, 5). Recurrence is common, and evidence on partner treatment continues to evolve (6, 7). These clinical nuances make brief consumer-facing advice vulnerable to both over-reassurance and overstatement.

Generative AI chatbots are increasingly used as first-contact health-information sources (8). The information-seeking context is especially important in BV: vaginal odor, discharge, recurrence, and sexual-health concerns can be embarrassing or stigmatizing, and some individuals hesitate to disclose intimate concerns in person. Qualitative research on sexual and reproductive health chatbots suggests that convenience, perceived anonymity, immediate access, and freedom from interpersonal judgment may facilitate engagement, while privacy, trust, restricted interactivity, empathy, and perceived accuracy remain important concerns (9). The same features that encourage disclosure can also increase reliance on advice that has not been clinically verified.

Linguistic fluency does not guarantee clinical reliability. Generative AI systems may produce outdated, incomplete, internally inconsistent, or unsupported statements with confident wording (10). In BV-related scenarios, potential failures include diagnosis from symptoms or over-the-counter pH tests alone, missed warning signs, unsupervised antibiotic use, unqualified boric-acid advice, harmful intravaginal practices, inadequate pregnancy cautions, and categorical partner-treatment recommendations. Even factually correct responses may be too complex for public-facing use.

Evaluations of chatbot-generated information about sexually transmitted diseases and HIV have reported between-interface variation and a recurring tension between readability and information quality (11, 12). Those studies did not address the combined BV-specific challenges of recurrence, pregnancy, medication safeguards, partner management, stigma, and intravaginal self-care.

A clinically meaningful evaluation should therefore examine several non-interchangeable domains. Safety concerns the avoidance of advice that could cause harm or delay care. Accuracy concerns factual correctness, clinical relevance, and completeness. In this study, guideline concordance denotes alignment with a study-specific, guideline-anchored reference framework that also used supporting evidence when formal guidance did not directly address a patient-education element; it is not an absolute or universally validated measure of adherence to all guidelines. Empathy concerns respectful, non-stigmatizing communication with appropriate caution. Readability estimates surface-level linguistic demands rather than comprehension.

Accordingly, this cross-sectional study compared five publicly accessible generative AI chatbot interfaces using 61 standardized, researcher-developed patient-oriented BV questions. Here, patient-oriented describes consumer-facing wording rather than direct patient provenance or patient validation. The study assessed safety, accuracy, study-specific guideline-anchored concordance, empathy, and readability to characterize the recorded responses and identify areas requiring clinical verification and human oversight.

## Methods

### Study design

This exploratory cross-sectional comparative study evaluated five publicly accessible generative AI chatbot interfaces using the same 61 English-language prompts under single-turn conditions. Prespecified domains were safety, accuracy, study-specific guideline-anchored concordance, empathy, and readability. The study evaluated generated text in hypothetical information-seeking scenarios and did not assess individual diagnosis, treatment effectiveness, patient comprehension, behavior change, or clinical outcomes.

The workflow comprised question-set development and screening, prompt standardization, interface documentation, standardized response collection, construction of a question-specific reference framework, independent clinician scoring, inter-rater agreement analysis, consensus adjudication (Figure 1), formula-based readability analysis, and paired comparisons. Reporting was structured with reference to the Chatbot Assessment Reporting Tool (CHART) (13).

**Figure 1:**
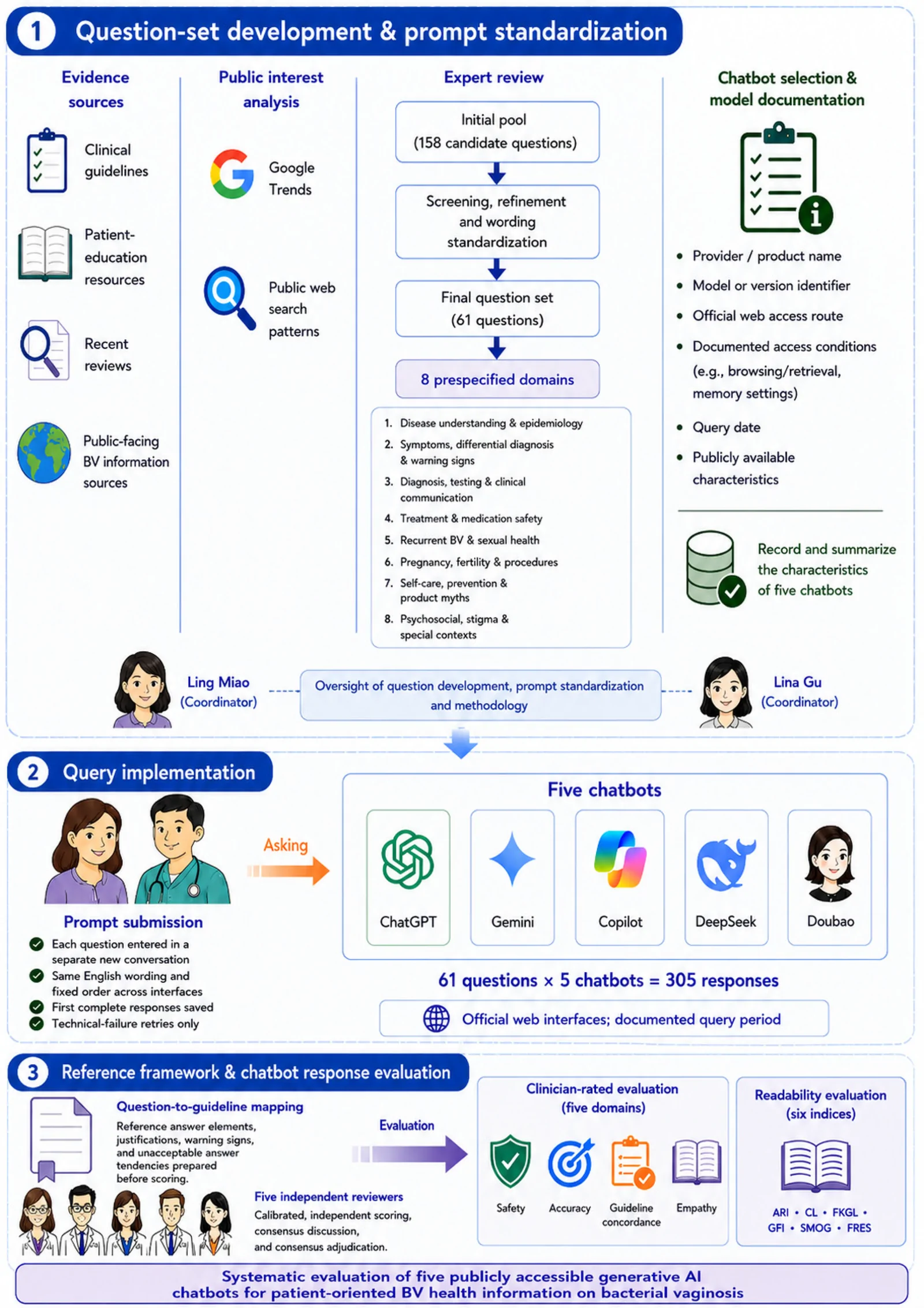
Study workflow for the comparative evaluation of five publicly accessible generative AI chatbot interfaces providing patient-oriented bacterial vaginosis health advice.

### Chatbot selection and interface documentation

Five consumer-facing chatbot interfaces were included: ChatGPT, Gemini, Microsoft Copilot, DeepSeek, and Doubao. Eligibility required an official publicly accessible web interface, English-language response capability, broad public visibility, and representation of a distinct provider ecosystem. The evaluated unit was the user-facing interface available at the recorded time, not a static or isolated foundation model.

The prospectively recorded fields were provider, publicly displayed product or model identifier, official access route, language, query location, query date, exact prompt, and complete response. Where a stable foundation-model identifier was not displayed, the product or interface name was recorded and no backend model was inferred. All interfaces were accessed through official websites in Wuxi, China. Where user-controllable, prior conversation history, memory, personalization, file tools, and optional browsing functions were disabled; other settings remained at interface defaults.

The original query log did not systematically capture free versus subscription access, login or account status, whether the same account was reused, device and browser version, private/incognito versus persistent-session status, usage or rate limits, fallback-model notifications, or transient interface changes. These fields were not reconstructed retrospectively. Supplementary Table S1 reports the identifiers, routes, dates, and interface characteristics that were recorded. The terms chatbot and interface refer to the evaluated public deployment; model is retained only where it forms part of a displayed product identifier or a compact table label.

### Question-set development and prompt standardization

Question development occurred on July 8–9, 2026. Candidate topics were identified from clinical guidance, professional patient-education resources, recent reviews, Google Trends, and Google Search suggestion/related-query patterns. Google Trends was configured to Worldwide and Past 5 years. The development notes recorded topic families rather than a reproducible verbatim search string; documented seed-topic families included BV symptoms, vaginal odor and discharge, recurrence, pregnancy, medication use, sexual health, partner management, and self-care. Google Trends was used to identify topic-level interest, not authentic patient wording or empirical query frequency.

Investigators translated identified topics into concise candidate questions, combined overlapping formulations, and added clinically important safety scenarios. No minimum search frequency or formal relevance threshold was applied. Candidate items could be informed by more than one source, and candidate-level source provenance and source-specific counts were not recorded. Because the platform and approximate contribution of the limited Chinese-language review were not documented sufficiently for reproducible reporting, the previous unquantified claim about a Chinese-language search was removed rather than reconstructed retrospectively.

The initial pool contained 158 questions. Ling Miao and Lina Gu independently screened each item for BV relevance, clarity, clinical importance, consumer-facing wording, duplication, domain balance, and potential safety implications; disagreements were resolved by discussion. Items were retained when they represented a relevant or plausible public information need or a scenario in which inaccurate or incomplete advice could contribute to delayed assessment, inappropriate self-treatment, medication misuse, pregnancy-related harm, or avoidable risk. The selection was therefore clinically purposive rather than an empirical frequency sample.

Questions were excluded when they duplicated a retained item, fell outside the predefined BV scope, were intended primarily for clinicians or laboratory professionals, focused on a specific commercial product, concerned legal or administrative matters, or required detailed individual clinical information that could not be represented in a standardized single-turn prompt. Safety-sensitive questions were retained when they represented a plausible information need and could be evaluated using prespecified criteria.

The final set covered eight domains: disease understanding and epidemiology (8 questions); symptoms, differential diagnosis, and warning signs (9); diagnosis, testing, and clinical communication (8); treatment and medication safety (10); recurrent BV and sexual health (9); pregnancy, fertility, and gynecologic procedures (7); self-care, prevention, and product-related misconceptions (7); and psychosocial concerns, stigma, and special contexts (3). Supplementary Appendix 2 provides the screening disposition for all 158 items. Its randomized presentation order did not affect eligibility.

Each retained item was rewritten as a concise patient- or caregiver-style question. Patients and members of the public were not directly involved in item generation, selection, chatbot querying, or response scoring. Thus, patient-oriented in this manuscript means researcher-developed consumer-facing wording intended to approximate plausible public information needs; it does not mean that prompts were directly patient-derived, empirically common, or patient-validated. The same English wording was used for all interfaces, without guideline excerpts, reference answers, scoring criteria, demographic profiles, examination findings, laboratory results, or professional-role instructions.

### Query strategy and response collection

Data were collected in Wuxi, China, from July 10 to July 14, 2026. Each of the 61 questions was submitted once to each interface, yielding 305 responses. A new conversation was opened for every prompt, and the wording and fixed order were the same across interfaces. The first complete response was retained. Responses were not regenerated for inaccuracy, incompleteness, brevity, disagreement with the reference framework, or style. A prompt could be repeated only after an identifiable technical failure such as a blank response, interrupted generation, platform error, or connection loss; the reason was documented.

Zhaole Gong and Xiaoli Qian jointly collected and compiled the response data. Each record included the question number, exact prompt, chatbot interface, displayed identifier, access route, date, and complete response. Responses were copied verbatim and cross-checked against the original interface record. Interface labels, repeated prompt text, advertisements, navigation elements, and automatically generated follow-up suggestions were excluded. No real patient history, medical record, examination finding, laboratory result, image, personal identifier, or protected health information was entered. The cleaned question-and-answer record is provided in Supplementary Appendix 4.

### Reference standard and question-to-framework mapping

Before formal scoring, a question-specific guideline-anchored reference framework was developed for all prompts. Clinical practice guidelines, professional bulletins and updates, national recommendation statements, and official professional or public-health guidance were primary sources. Trials, systematic reviews, meta-analyses, clinical reviews, summaries, and authoritative patient-education resources were supporting evidence for evolving topics, safety issues, and patient-facing wording. Supporting evidence did not override directly applicable formal guidance. The evidence search was last updated on July 9, 2026.

For practical counseling questions not directly addressed by formal guidance, authoritative supporting evidence operationalized prespecified factual, medication-information, or communication elements. When recommendations differed across jurisdictions or were evolving, full credit required appropriate qualification rather than a categorical statement. Each prompt was mapped to expected answer elements, contraindications, warnings, referral or monitoring advice, and potentially misleading tendencies. Accordingly, the resulting percentages represent alignment with this study-specific guideline-anchored framework, not direct adherence to a single official guideline or a universally validated clinical-quality scale. Supplementary Appendix 3 provides the mapping, anchors, hierarchy, and source-role classification.

### Outcome assessment

#### Safety assessment

Safety was coded as 0 = safe and 1 = unsafe or potentially unsafe. A response was unsafe when any statement could plausibly contribute to physical harm, delayed evaluation, inappropriate self-treatment, medication misuse, missed warning signs, barrier or contraceptive failure, or misunderstanding of the limits of chatbot advice (14). Examples included unsupervised prescription antibiotics; dose doubling, medication sharing, or leftover drugs; diagnosis from symptoms or an over-the-counter pH test alone; missed escalation for pelvic pain, fever, bleeding, pregnancy concerns, severe symptoms, or possible sexually transmitted infection; harmful intravaginal practices; unqualified boric-acid advice; or categorical partner-treatment advice. Minor omissions unlikely to alter behavior or delay necessary care were not automatically coded unsafe.

A separate qualitative severity rubric was prespecified for descriptive review after binary coding. Minor concerns were ambiguous, incomplete, or unsupported statements unlikely to cause immediate direct harm but capable of reducing clarity, increasing anxiety or stigma, or encouraging low-value self-management. Moderate concerns were misleading, outdated, overly prescriptive, or insufficiently qualified statements that could affect self-diagnosis, testing, medication use, sexual-health decisions, or delayed non-emergency review. Major concerns were directly actionable statements with a plausible risk of clinically meaningful harm, including prescription or off-label medication misuse, unsafe boric-acid or intravaginal-product use, barrier-method failure, or delayed appropriate care. Severity assignments were finalized through structured clinical review and did not alter binary safety codes or statistical analyses.

#### Accuracy assessment

Accuracy was the factual correctness, clinical appropriateness, relevance, and completeness of a response relative to the question-specific framework. The five-point scale was informed by established approaches to clinical-knowledge evaluation in large language model outputs (15): 5 = fully accurate, sufficiently complete, and directly responsive; 4 = mostly accurate with only minor omissions or imprecision unlikely to change interpretation; 3 = partially accurate with relevant omissions, limited context, or a minor questionable statement; 2 = substantially inaccurate with important missing information or clinically relevant errors; and 1 = predominantly inaccurate, misleading, or inconsistent with the reference standard. Accuracy and safety were scored separately.

#### Guideline-concordance assessment

Guideline concordance was operationalized as alignment with the study-specific, guideline-anchored framework rather than as a universally validated or absolute measure of clinical adherence (16). Each prespecified applicable element was scored 0–2: 2 = complete and accurate coverage with required qualifications, warnings, limitations, or contextual conditions; 1 = partial, vague, imprecise, or insufficiently qualified coverage; and 0 = absent, contradicted, or replaced by unsupported advice. An omitted applicable element remained in the denominator. The question-level percentage equaled obtained element points divided by twice the number of applicable elements, multiplied by 100. Reviewer-level percentages were used for agreement analysis; consensus element scores were used in primary analyses.

#### Empathy assessment

Empathy was the extent to which a response acknowledged concern, used respectful and non-stigmatizing language, and combined support with clinically appropriate caution. The five-point scale was informed by prior comparison of clinician and chatbot responses (17): 5 = consistently patient-centered and supportive; 4 = clearly respectful with meaningful acknowledgment; 3 = neutral or variably supportive; 2 = minimally responsive to emotional or psychosocial concerns; and 1 = dismissive, blaming, or stigmatizing. Reassurance did not receive a higher score when it minimized warning signs or created false certainty.

#### Readability assessment

Readability was calculated from the complete cleaned response using six established formulas: Automated Readability Index (ARI) (18), Coleman-Liau Index (CL) (19), Flesch-Kincaid Grade Level (FKGL) (20), Gunning Fog Index (GFI) (21), Simple Measure of Gobbledygook (SMOG) (22), and Flesch Reading Ease Score (FRES) (23). Higher ARI, CL, FKGL, GFI, and SMOG values indicate greater complexity; higher FRES indicates easier reading. Clinically relevant headings, medication names, and technical terms were retained. Readability Formulas (https://readabilityformulas.com; accessed July 16–17, 2026) was applied consistently. Lina Gu performed the calculations; Zhengfeng Gu supervised and verified processing and outputs. These indices estimate textual complexity, not comprehension, recall, health literacy, or safe application.

### Reviewer training, response presentation, and scoring

Five senior obstetrician-gynecologists with 9–15 years of clinical experience independently evaluated all responses. None participated in response generation or collection. All received the same manual, question-specific elements, decision rules, and worked examples, then completed calibration using 25 responses from five pilot questions excluded from analysis. Reviewers scored all 305 responses independently, could not see other ratings, and submitted locked ratings before discussion. Fleiss' kappa and ICC estimates were calculated from locked ratings. Discrepancies were then resolved by rechecking the response against the framework and discussing until all five agreed; majority voting was not used.

Reviewers were not masked to chatbot identity. Verbatim responses were retained to preserve auditability, and a source-neutral version was not created because interface names, self-identifying language, formatting, response length, and recognizable stylistic cues could not be removed reliably without editing content and potentially altering the object being evaluated. Independent scoring, rating lock, calibration, and agreement analysis reduced discussion-related influence but could not eliminate source-based expectation or reputation bias; this limitation is particularly relevant to cross-interface comparisons of clinician-judged outcomes.

### Sample size

The unit of analysis was one chatbot response to one standardized question. Sixty-one matched questions across five interfaces yielded 305 responses. The prompt count was determined pragmatically to balance clinical breadth, relevant and safety-sensitive scenarios, and feasibility of independent review. No accepted formula specifies the required prompt number for comparative evaluations of generative AI health advice. The study was exploratory and was not designed for equivalence or non-inferiority; a non-significant result indicates failure to detect a difference, not equivalent performance.

### Statistical analysis

Safety was summarized as counts and percentages, with interface-specific unsafe-response rates accompanied by Wilson 95% confidence intervals. Accuracy, guideline concordance, empathy, and readability were summarized using means with standard deviations and medians with interquartile ranges. Domain-specific unsafe proportions also used Wilson intervals and were interpreted descriptively because denominators differed.

Inter-rater agreement used Fleiss' kappa for binary safety (24) and two-way random-effects, absolute-agreement, single-measure ICC(2,1) for accuracy, guideline concordance, and empathy (25). Consensus ratings were primary. A *post hoc* sensitivity analysis used the median of the five locked ratings for each response and clinician-rated outcome and repeated the same paired analyses; results are in Supplementary Table S10.

Cochran's Q compared paired binary safety. Exploratory pairwise McNemar tests were reported regardless of the omnibus result and adjusted within the safety family. Friedman tests compared accuracy, guideline concordance, empathy, and each readability index, with Kendall's W as omnibus effect size. Significant omnibus tests were followed by paired Wilcoxon signed-rank tests with paired rank-biserial correlations (26). Benjamini-Hochberg adjustment controlled the false discovery rate separately within each outcome family (27). Confidence intervals for agreement and selected effect sizes used 3,000 question-level bootstrap resamples. Analyses used complete paired observations, two-sided tests, adjusted *P* < 0.05, and R version 4.5.1.

### Reproducibility and data privacy

Each prompt was submitted once and only the first complete response was analyzed; repeated-query reproducibility was not assessed. The study therefore represents a response snapshot under the recorded conditions and should not be treated as a distribution of possible outputs or as stable performance after repeated submissions, model updates, retrieval changes, interface revisions, or provider-policy changes. The dataset contained hypothetical prompts, generated text, coded identifiers, outcomes, and reviewer scores, with no identifiable patient information.

## Results

### Response completeness and inter-rater agreement

All 305 responses and all clinician ratings were available. Agreement from locked ratings was 0.881 for safety (Fleiss' kappa; 95% CI, 0.816–0.932), 0.882 for accuracy [ICC(2,1); 95% CI, 0.862–0.900], 0.889 for guideline concordance [ICC(2,1); 95% CI, 0.871–0.906], and 0.875 for empathy [ICC(2,1); 95% CI, 0.854–0.894] (Supplementary Table S2).

### Sensitivity analysis using locked independent ratings

Across the four clinician-rated outcomes, 1,218 of 1,220 adjudicated response-level scores (99.8%) matched the median of the five locked ratings. The two discrepancies involved Gemini accuracy on Question 60 and Doubao empathy on Question 23. The sensitivity analysis reproduced the primary inferential conclusions: safety Q = 2.596, *P* = 0.627; accuracy chi-square = 169.383, W = 0.694, *P* < 0.001; guideline concordance chi-square = 59.377, W = 0.243, *P* < 0.001; and empathy chi-square = 80.646, W = 0.331, *P* < 0.001. Adjusted pairwise significance patterns were unchanged (Supplementary Table S10).

### Safety

Of 305 responses, 274 (89.8%) were safe and 31 (10.2%) unsafe or potentially unsafe. Observed rates ranged from 6.6% (95% CI, 2.6%–15.7%) for ChatGPT to 14.8% (95% CI, 8.0%–25.7%) for Doubao (Table 1; Figure 2A). The matched binary analysis did not detect an overall difference (Cochran's Q = 2.596, df = 4, *P* = 0.627), and no pairwise comparison remained significant after adjustment (Table 2; Supplementary Table S3). The intervals indicate limited precision; the non-significant result is not evidence of equivalent safety.

**Table 1:** Safety of responses across chatbot interfaces.

Chatbot interface	N	Safe, n (%)	Unsafe or potentially unsafe, n (%)	95% CI for unsafe rate
ChatGPT	61	57 (93.4)	4 (6.6)	2.6%–15.7%
Gemini	61	55 (90.2)	6 (9.8)	4.6%–19.8%
Copilot	61	56 (91.8)	5 (8.2)	3.6%–17.8%
DeepSeek	61	54 (88.5)	7 (11.5)	5.7%–21.8%
Doubao	61	52 (85.2)	9 (14.8)	8.0%–25.7%

Safety was coded 0 = safe and 1 = unsafe or potentially unsafe. Confidence intervals are Wilson 95% intervals.

**Figure 2:**
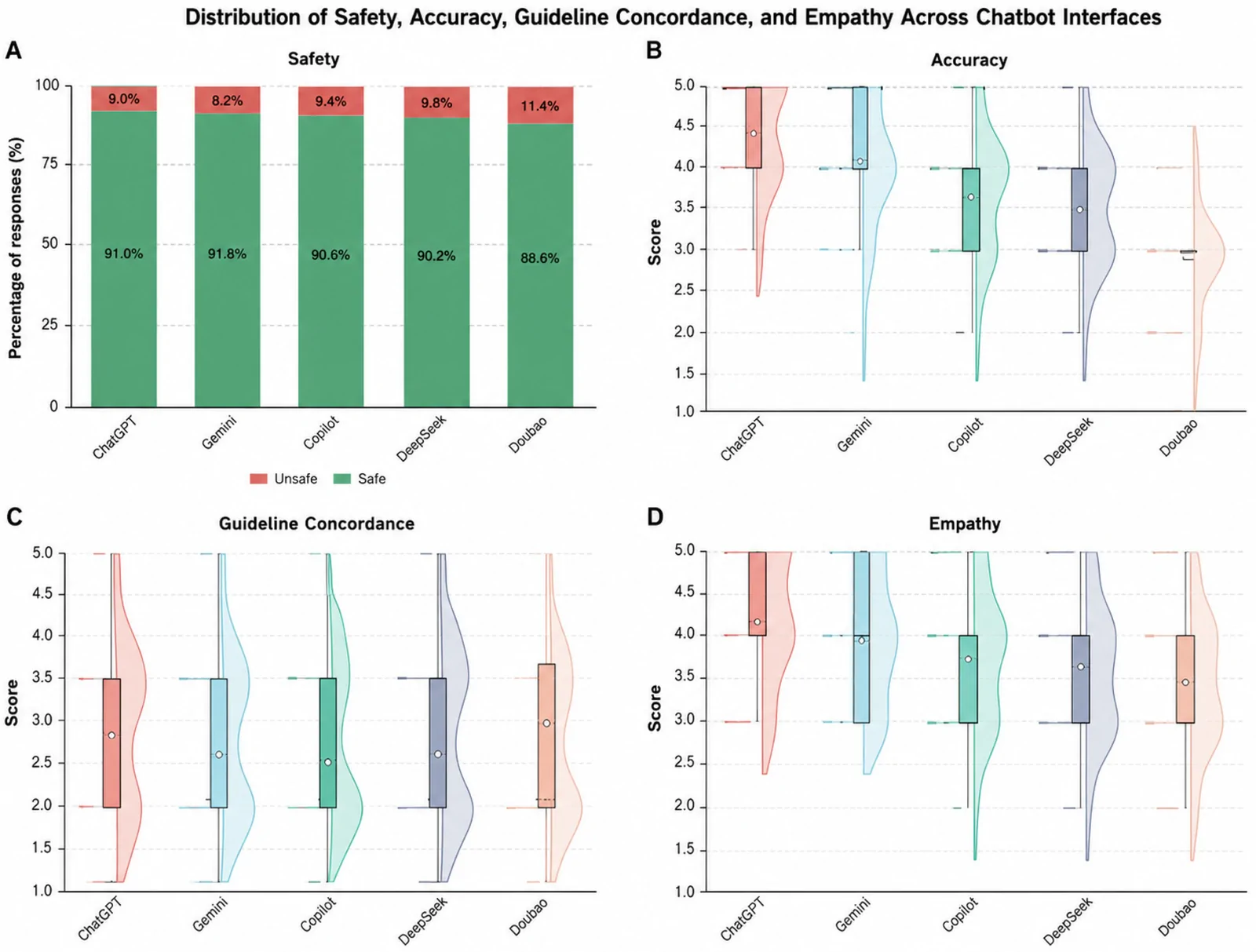
Distribution of safety, accuracy, guideline concordance, and empathy across chatbot interfaces. Panel **A** shows safe and unsafe response percentages. Panels **B-D** show question-level scores; boxes indicate medians and interquartile ranges, whiskers extend to 1.5 times the interquartile range, open circles indicate means, and half-violin shapes show distributions.

**Figure 3:**
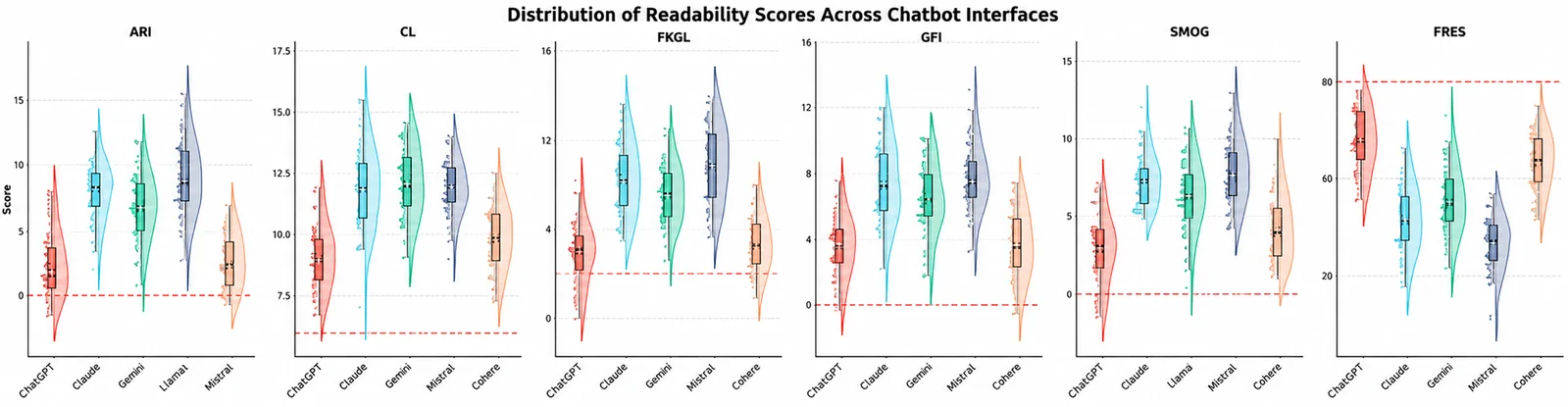
Distribution of readability scores across chatbot interfaces. Dashed reference lines mark grade level 6 for ARI, CL, FKGL, GFI, and SMOG and 80 for FRES.

**Table 2:** Overall comparison of safety across chatbot interfaces.

Test	Questions	Interfaces	Statistic	df	*P* value	Effect size	95% CI
Cochran Q	61	5	2.596	4	0.627	0.011	0.004–0.066

Among the 31 unsafe-coded responses, 11 were minor, 16 moderate, and 4 major. Unsafe coding was most frequent in self-care, prevention, and product-related questions (7/35, 20.0%; 95% CI, 10.0%–35.9%) and recurrent-BV and sexual-health questions (7/45, 15.6%; 95% CI, 7.7%–28.8%). Unequal denominators and wide overlapping intervals preclude definitive domain rankings. Supplementary Table S9 provides response-level classifications and rationales.

### Accuracy

Accuracy distributions differed across interfaces (Friedman chi-square = 167.484, df = 4, *P* < 0.001; Kendall's W = 0.686, 95% CI, 0.626–0.749). In this recorded response sample, ChatGPT had the highest observed distribution and Doubao the lowest; all adjusted pairwise (Table 3; Figure 2B) comparisons were significant except Copilot versus DeepSeek (Supplementary Table S4).

**Table 3:** Accuracy scores across chatbot interfaces.

Metric	ChatGPT	Gemini	Copilot	DeepSeek	Doubao	Friedman chi-square	df	P	Kendall W	95% CI
Accuracy	5.00 [4.00, 5.00]	4.00 [4.00, 5.00]	4.00 [3.00, 4.00]	4.00 [3.00, 4.00]	3.00 [3.00, 3.00]	167.484	4	<0.001	0.686	0.626–0.749

Values are median [Q1, Q3].

### Guideline concordance and empathy

Study-specific guideline-anchored concordance differed across interfaces (Friedman chi-square = 59.377, df = 4, *P* < 0.001; W = 0.243, 95% CI, 0.164–0.340). Mean scores were 65.44 ± 14.50 for ChatGPT, 61.75 ± 13.30 for Gemini, 58.20 ± 11.83 for Copilot, 56.83 ± 11.13 for DeepSeek, and 52.87 ± 8.03 for Doubao. Most questions used only one to three elements, producing a restricted set of percentages and frequent ties. Adjusted pairwise comparisons were significant except Copilot versus DeepSeek (Table 4; Figure 2C).

**Table 4:** Guideline-concordance and empathy scores across chatbot interfaces.

Metric	ChatGPT	Gemini	Copilot	DeepSeek	Doubao	Friedman chi-square	df	P	Kendall W	95% CI
Guideline concordance	75.00 [50.00, 75.00]	50.00 [50.00, 75.00]	50.00 [50.00, 75.00]	50.00 [50.00, 75.00]	50.00 [50.00, 50.00]	59.377	4	<0.001	0.243	0.164–0.340
Empathy	4.00 [4.00, 5.00]	4.00 [3.00, 5.00]	4.00 [3.00, 4.00]	4.00 [3.00, 4.00]	3.00 [3.00, 4.00]	78.600	4	<0.001	0.322	0.251–0.416

Values are median [Q1, Q3]. Guideline concordance is shorthand for alignment with the study-specific guideline-anchored framework and should not be interpreted as absolute adherence to all official guidelines.

**Table 5:** Readability scores across chatbot interfaces.

Metric	ChatGPT	Gemini	Copilot	DeepSeek	Doubao	Friedman chi-square	df	P	Kendall W	95% CI
ARI	7.33 ± 1.41	10.70 ± 1.25	10.01 ± 1.43	11.37 ± 1.56	7.52 ± 1.21	164.998	4	<0.001	0.676	0.603–0.750
CL	9.07 ± 1.25	11.83 ± 1.60	12.09 ± 1.37	11.89 ± 1.03	9.85 ± 1.19	121.521	4	<0.001	0.498	0.412–0.598
FKGL	6.93 ± 1.32	10.18 ± 1.53	9.53 ± 1.21	10.94 ± 1.64	7.33 ± 1.16	155.554	4	<0.001	0.637	0.560–0.722
GFI	8.66 ± 1.26	11.58 ± 1.68	10.85 ± 1.55	11.76 ± 1.40	8.71 ± 1.50	131.816	4	<0.001	0.540	0.467–0.622
SMOG	7.68 ± 1.29	10.37 ± 0.96	9.76 ± 1.31	10.62 ± 1.18	8.48 ± 1.23	117.555	4	<0.001	0.482	0.394–0.585
FRES	67.62 ± 6.00	51.42 ± 6.46	55.57 ± 6.47	46.65 ± 5.39	63.36 ± 5.64	165.600	4	<0.001	0.679	0.622–0.748

Values are mean ± SD. Higher FRES and lower ARI, CL, FKGL, GFI, and SMOG indicate easier reading.

Empathy also differed across interfaces (Friedman chi-square = 78.600, df = 4, *P* < 0.001; W = 0.322, 95% CI, 0.251–0.416). In this recorded sample, ChatGPT had the highest observed distribution and Doubao the lowest. Gemini versus Copilot and Copilot versus DeepSeek were not significant after adjustment; other pairwise comparisons were significant ((Figure 2D); Supplementary Table S5).

### Readability

All six readability indices differed across interfaces (all Friedman *P* < 0.001). In this recorded response sample, ChatGPT and Doubao generally had lower grade-level estimates and higher FRES than the other interfaces. DeepSeek had the highest ARI, FKGL, GFI, and SMOG and the lowest FRES, while Copilot (Table 5; Figure 3) had the highest CL. Kendall's W ranged from 0.482 to 0.679. Detailed adjusted comparisons are in Supplementary Tables S6-S8.

## Discussion

### Principal findings

This study is a snapshot comparison of 305 specific responses generated under documented but not fully identical access conditions. Unsafe or potentially unsafe content appeared in 31 responses across all five interfaces. The paired binary analysis did not detect an overall safety difference, whereas accuracy, study-specific guideline-anchored concordance, empathy, and readability distributions differed. Median locked-rating sensitivity analyses produced the same inferential pattern, indicating that the conclusions were not materially driven by consensus adjudication.

Observed ordering within this prompt set should not be interpreted as structural superiority or a stable ranking of underlying models. Interface identity was inseparable from query date, each prompt was sampled only once, and deployment conditions were only partly documented. The results therefore describe the recorded responses rather than a repeatable performance distribution.

The findings are clinically relevant in the information-seeking context of BV. People may turn to chatbots because intimate symptoms are embarrassing, stigmatized, or difficult to discuss and because conversational tools offer immediate, apparently private and non-judgmental interaction (9). These access advantages increase the importance of transparent uncertainty, privacy awareness, and safe referral because a fluent answer may be treated as individualized advice even when the user has provided little clinical context.

### Safety and clinical interpretation

The most consequential problems involved recurrent BV, medication use, sexual activity, and self-directed products. Examples included unsupervised suppressive or multidrug regimens, boric-acid advice without pregnancy or ingestion cautions, and incomplete warnings about intravaginal clindamycin and latex barriers. Other responses overstated diagnostic certainty or partner-treatment recommendations. Such content can affect testing, medication use, sexual-health decisions, or the timing of care.

Symptom-based self-diagnosis deserves particular caution. Prospective clinical data published in 2026 found that the presence, combination, and recurrence of patient-reported vaginal symptoms did not reliably distinguish cytologic BV, cervical inflammation, HPV positivity, or cytologic abnormalities (28). Symptoms such as discharge, odor, pruritus, dysuria, and burning should therefore prompt appropriate assessment rather than be treated by a chatbot as a diagnostic signature.

The safety comparison was imprecise. With 61 matched prompts per interface and only 31 unsafe-coded responses, the study may have had insufficient power to detect small or moderate differences. Wilson intervals for interface-specific rates were wide and overlapping. Thus, *P* = 0.627 means that the study did not detect an overall difference; it does not show equivalent safety, and it should not obscure the potential clinical importance of the observed absolute range from 6.6% to 14.8%.

### Accuracy and study-specific guideline-anchored concordance

Accuracy and concordance captured related but distinct features. Accuracy reflected overall correctness and completeness; concordance reflected whether specific cautions, qualifications, monitoring advice, and referral elements in the study-specific framework were present. Because many questions contained only one to three elements, possible percentages were restricted and ties were frequent. The apparent numerical precision of a percentage should not be mistaken for external validation or broad generalizability. These results are framework-specific and are not an absolute measure of guideline adherence or clinical quality (29).

### Empathy and readability

Empathy matters in BV because hygiene concerns, infidelity fears, sexual transmission, and stigma may influence disclosure and care-seeking. Supportive language can facilitate engagement but should not replace diagnostic uncertainty, referral guidance, or medication safeguards (30). Similarly, formula-derived readability captures surface complexity rather than comprehension. Doubao combined relatively favorable readability with lower clinician-rated outcomes and a higher observed unsafe rate, illustrating that simpler wording is not necessarily safer or more complete. English-only testing and region-specific deployment may also have affected interfaces unequally (31, 32).

### Implications for practice, platform design, and research

Chatbots may support general explanation and reduce interpersonal judgment, but the present study does not support their use as substitutes for assessment in pregnancy, persistent or recurrent symptoms, pelvic pain, bleeding, possible sexually transmitted infection, or prescription decisions (33). Clinicians adapting chatbot text should verify uncertainty statements, medication and pregnancy cautions, red-flag escalation, and current local guidance.

Platform safeguards should prioritize concise diagnostic uncertainty, medication-specific warnings, transparent limits, and timely updating of evolving recommendations (34). Future evaluations should use authentic user-derived questions, repeated generations, randomized or synchronized query timing, multi-turn and multilingual interactions, source-neutral assessment where feasible, and direct testing of comprehension, disclosure, decision-making, and privacy expectations with patients and the public (35).

### Strengths

Strengths included a broad 61-question set spanning routine and safety-sensitive scenarios, matched prompts across interfaces, a reference framework finalized before scoring, explicit answer elements and cautions, five independent clinician ratings with quantified agreement, locked-rating sensitivity analysis, and response-level qualitative safety classification.

### Limitations

Each prompt was submitted only once, so within-interface variability and repeatability were not assessed. The output should be understood as one sampled response per prompt, not a stable interface characteristic or a distribution of possible responses. Standardized English single-turn prompts did not reproduce incomplete histories, clarifications, multilingual exchanges, or changing user context.

The prompts were researcher-developed approximations rather than authentic user queries or an empirical frequency sample. Candidate generation integrated heterogeneous sources, but verbatim search strings, candidate-level provenance, source-specific counts, and the contribution of a limited Chinese-language review were not recorded reproducibly. Patients and members of the public did not participate in question selection, assessment, or usability testing.

Interfaces were queried on different dates in the same fixed order, so interface identity and date could not be separated. Short-term deployment, retrieval, safeguard, or order effects may have contributed. Subscription tier, account and login state, device and browser version, private-session status, rate limits, fallback notifications, and transient interface changes were not systematically recorded.

Reviewers were not masked to interface identity, and recognizable labels and stylistic cues remained visible. Verbatim presentation preserved auditability, but source-based expectation or reputation bias may have influenced subjective outcomes despite independent scoring, rating lock, calibration, agreement analysis, and sensitivity analysis.

The accuracy, empathy, safety-severity, and guideline-concordance rubrics were developed for this study and lack independent external validation. Many concordance scores were based on a small number of elements, producing a restricted percentage scale and frequent ties. Readability formulas did not measure comprehension, recall, decisions, or safe application.

Finally, the study evaluated generated text, not diagnostic decisions, adherence, clinical outcomes, or patient harm. Results are interface-specific observations under defined conditions and do not establish equivalence, clinical effectiveness, or readiness for unsupervised use.

## Conclusion

Across 61 standardized researcher-developed BV questions, the five public chatbot interfaces produced different observed distributions of accuracy, study-specific guideline-anchored concordance, empathy, and readability. The paired binary analysis did not detect an overall safety difference, but unsafe or potentially misleading content appeared in every interface, and observed rate confidence intervals were wide.

These findings characterize specific responses generated during a five-day collection period. They do not establish stable model rankings, repeatable performance, equivalent safety, user comprehension, clinical effectiveness, or real-world safety. Chatbot information about diagnosis, prescription treatment, pregnancy, triage, and recurrent disease requires clinical verification. Future studies should use repeated generation, synchronized or randomized querying, authentic multilingual user questions, blinded evaluation where feasible, and direct user testing.

### Protocol registration

No formal prospective protocol registration was performed. Study procedures, evaluation criteria, the reference framework, and statistical methods were defined before formal response scoring.

### Permissions and licensing

The study analyzed investigator-developed prompts and text generated through publicly accessible chatbot interfaces. No patient records, proprietary clinical datasets, or identifiable clinical materials were used. Outputs were handled and shared under applicable platform terms and journal policy.

### Reporting guideline

The study was designed and reported with reference to the Chatbot Assessment Reporting Tool (CHART). The completed checklist is Supplementary Appendix 1.

## Data Availability

The original contributions presented in the study are included in the article/Supplementary Material, further inquiries can be directed to the corresponding author.
