# Supplementary material for: A comparative cross-sectional evaluation of generative AI chatbots for patient-oriented bacterial vaginosis health advice: safety, accuracy, guideline concordance, empathy, and readability
Source: Front Reprod Health. 2026 Sep 17;8:1967818. doi: 10.3389/frph.2026.1967818 (PMC13627438; doi:10.3389/frph.2026.1967818)
Supplement: Supplementary file 4 [file Datasheet5.pdf]

Supplementary Tables

Supplementary Table S1. Model identifiers and access details

Chatbot	Provider	Model/version identifier	Access route	Source availability	Model type evaluated	Language setting	Query date	Notes
ChatGPT-4.0	OpenAI	ChatGPT-4.0	Official ChatGPT web interface	Proprietary / closed-source deployed interface	User-facing deployed chatbot	English	10July 2026	Complete training data, model weights, and deployment parameters were not publicly available
Gemini 2.5 Pro	Google / Google DeepMind	Gemini 2.5 Pro	Official Gemini web interface	Proprietary / closed-source deployed interface	User-facing deployed chatbot	English	11July 2026	Complete training data, model weights, and deployment parameters were not publicly available
Microsoft Copilot	Microsoft	GPT-5-based Copilot deployment	Official Microsoft Copilot web interface	Proprietary / closed-source deployed interface	User-facing deployed chatbot	English	12July 2026	Underlying deployment details may vary and were recorded according to the interface available at query
Doubao	ByteDance	Doubao-Seed-1.6	Official Doubao web interface	Proprietary / closed-source deployed interface	User-facing deployed chatbot	English	13July 2026	Complete training data, model weights, and deployment parameters were not publicly available
DeepSeek-V3.2-Exp	DeepSeek	DeepSeek-V3.2-Exp	Official DeepSeek web interface	Deployed chatbot interface; full deployment details not publicly available	User-facing deployed chatbot	English	14July 2026	Evaluation focused on the deployed web chatbot rather than locally hosted model weights

Note: The exact subscription tier and the status of optional functions that were not user-controllable were not completely documented in the original query log and were not inferred retrospectively. Where available, memory, personalization, file tools, and optional browsing functions were disabled; provider-default settings were otherwise retained.

Supplementary Table S2. Inter-rater agreement

Metric	Agreement index	Estimate	95% CI	P value	Interpretation	Complete items	No. of raters
Safety	Fleiss' kappa	0.881	0.816-0.932	<0.001	Good	305	5
Accuracy	ICC(2,1)	0.882	0.862-0.900	<0.001	Good	305	5

Metric	Agreement index	Estimate	95% CI	P value	Interpretation	Complete items	No. of raters
Guideline Concordance	ICC(2,1)	0.889	0.871-0.906	<0.001	Good	305	5
Empathy	ICC(2,1)	0.875	0.854-0.894	<0.001	Good	305	5

Note: Safety was assessed using Fleiss' kappa. Accuracy, Guideline Concordance, and Empathy were assessed using ICC(2,1).

Supplementary Table S3. Pairwise comparisons of safety

Comparison	N pairs	Safe rate difference, %	Safe rate difference 95% CI	Safe to unsafe, n	Unsafe to safe, n	Matched OR	Matched OR 95% CI	Unadjusted P	Adjusted P
ChatGPT vs Gemini	61	3.28	-6.56-13.11	6	4	1.50	0.42-5.32	0.752	1.000
ChatGPT vs Copilot	61	1.64	-8.20-11.48	5	4	1.25	0.34-4.66	1.000	1.000
ChatGPT vs DeepSeek	61	4.92	-4.92-16.39	7	4	1.75	0.51-5.98	0.546	1.000
ChatGPT vs Doubao	61	8.20	-1.64-18.03	8	3	2.67	0.71-10.05	0.228	1.000
Gemini vs Copilot	61	-1.64	-13.11-8.20	5	6	0.83	0.25-2.73	1.000	1.000
Gemini vs DeepSeek	61	1.64	-9.84-13.11	7	6	1.17	0.39-3.47	1.000	1.000
Gemini vs Doubao	61	4.92	-6.56-16.39	9	6	1.50	0.53-4.21	0.606	1.000
Copilot vs DeepSeek	61	3.28	-8.20-14.75	7	5	1.40	0.44-4.41	0.773	1.000
Copilot vs Doubao	61	6.56	-3.28-16.39	7	3	2.33	0.60-9.02	0.343	1.000
DeepSeek vs Doubao	61	3.28	-8.20-14.75	7	5	1.40	0.44-4.41	0.773	1.000

Note: Pairwise comparisons were performed using McNemar tests. Adjusted P values were calculated using the Benjamini-Hochberg method. A positive safe-rate difference indicates a higher safe-response rate for the first model.

Supplementary Table S4. Pairwise comparisons of accuracy

Metric	Comparison	N pairs	Mean difference	Mean difference 95% CI	Median difference	Median difference 95% CI	Rank-biserial correlation	Rank-biserial 95% CI	Unadjusted P	Adjusted P
Accuracy	ChatGPT vs Gemini	61	0.33	0.18-0.48	0.00	0.00-1.00	0.714	0.370-1.000	<0.001	<0.001
Accuracy	ChatGPT vs Copilot	61	0.75	0.59-0.90	1.00	1.00-1.00	0.959	0.864-1.000	<0.001	<0.001
Accuracy	ChatGPT vs DeepSeek	61	0.92	0.77-1.07	1.00	1.00-1.00	0.966	0.889-1.000	<0.001	<0.001
Accuracy	ChatGPT vs Doubao	61	1.51	1.38-1.66	2.00	1.00-2.00	1.000	1.000-1.000	<0.001	<0.001
Accuracy	Gemini vs Copilot	61	0.43	0.25-0.61	0.00	0.00-1.00	0.804	0.490-1.000	<0.001	<0.001
Accuracy	Gemini vs DeepSeek	61	0.59	0.44-0.74	1.00	0.00-1.00	0.948	0.818-1.000	<0.001	<0.001
Accuracy	Gemini vs Doubao	61	1.18	1.05-1.31	1.00	1.00-1.00	1.000	1.000-1.000	<0.001	<0.001
Accuracy	Copilot vs DeepSeek	61	0.16	0.02-0.31	0.00	0.00-0.00	0.417	0.000-0.751	0.052	0.052
Accuracy	Copilot vs Doubao	61	0.75	0.61-0.90	1.00	1.00-1.00	0.959	0.867-1.000	<0.001	<0.001
Accuracy	DeepSeek vs Doubao	61	0.59	0.46-0.70	1.00	0.00-1.00	1.000	1.000-1.000	<0.001	<0.001

Note: Paired Wilcoxon signed-rank tests were used. Positive mean differences and rank-biserial correlations indicate higher scores for the first model.

Supplementary Table S5. Pairwise comparisons of guideline concordance and empathy

Metric	Comparison	N pairs	Mean difference	Mean difference 95% CI	Median difference	Median difference 95% CI	Rank-biserial correlation	Rank-biserial 95% CI	Unadjusted P	Adjusted P
Guideline Concordance	ChatGPT vs Gemini	61	3.69	0.82-6.97	0.00	0.00-0.00	0.822	0.333-1.000	0.025	0.031
Guideline	ChatGPT vs	61	7.24	3.96-10.9	0.00	0.00-0.00	1.000	1.000-1.000	<0.001	0.001

Metric	Comparison	N pairs	Mean difference	Mean difference 95% CI	Median difference	Median difference 95% CI	Rank-biserial correlation	Rank-biserial 95% CI	Unadjusted P	Adjusted P
Concordance	Copilot			3						
Guideline Concordance	ChatGPT vs DeepSeek	61	8.61	4.92-12.30	0.00	0.00-0.00	1.000	1.000-1.000	<0.001	<0.001
Guideline Concordance	ChatGPT vs Doubao	61	12.57	8.74-16.39	0.00	0.00-25.00	0.938	0.795-1.000	<0.001	<0.001
Guideline Concordance	Gemini vs Copilot	61	3.55	0.82-6.56	0.00	0.00-0.00	0.667	0.127-1.000	0.032	0.036
Guideline Concordance	Gemini vs DeepSeek	61	4.92	2.05-8.20	0.00	0.00-0.00	0.857	0.505-1.000	0.003	0.005
Guideline Concordance	Gemini vs Doubao	61	8.88	5.74-12.16	0.00	0.00-0.00	1.000	1.000-1.000	<0.001	<0.001
Guideline Concordance	Copilot vs DeepSeek	61	1.37	-0.96-3.83	0.00	0.00-0.00	0.467	-0.267-1.000	0.200	0.200
Guideline Concordance	Copilot vs Doubao	61	5.33	2.87-8.20	0.00	0.00-0.00	0.867	0.538-1.000	<0.001	0.002
Guideline Concordance	DeepSeek vs Doubao	61	3.96	1.50-6.69	0.00	0.00-0.00	0.821	0.391-1.000	0.007	0.010
Empathy	ChatGPT vs Gemini	61	0.25	0.08-0.41	0.00	0.00-0.00	0.708	0.275-1.000	0.005	0.007
Empathy	ChatGPT vs Copilot	61	0.38	0.23-0.52	0.00	0.00-0.00	0.852	0.619-1.000	<0.001	<0.001
Empathy	ChatGPT vs DeepSeek	61	0.52	0.34-0.69	0.00	0.00-1.00	0.883	0.616-1.000	<0.001	<0.001

Metric	Comparison	N pairs	Mean difference	Mean difference 95% CI	Median difference	Median difference 95% CI	Rank-biserial correlation	Rank-biserial 95% CI	Unadjusted P	Adjusted P
Empathy	ChatGPT vs Doubao	61	0.74	0.61-0.87	1.00	1.00-1.00	1.000	1.000-1.000	<0.001	<0.001
Empathy	Gemini vs Copilot	61	0.13	-0.07-0.33	0.00	0.00-0.00	0.286	-0.192-0.723	0.246	0.246
Empathy	Gemini vs DeepSeek	61	0.28	0.15-0.43	0.00	0.00-0.00	0.895	0.667-1.000	<0.001	<0.001
Empathy	Gemini vs Doubao	61	0.49	0.31-0.69	0.00	0.00-1.00	0.782	0.498-1.000	<0.001	<0.001
Empathy	Copilot vs DeepSeek	61	0.15	-0.05-0.36	0.00	0.00-0.00	0.397	-0.133-0.859	0.149	0.166
Empathy	Copilot vs Doubao	61	0.36	0.20-0.54	0.00	0.00-0.00	0.778	0.500-1.000	<0.001	<0.001
Empathy	DeepSeek vs Doubao	61	0.21	0.03-0.38	0.00	0.00-0.00	0.506	0.053-0.918	0.031	0.039

Note: Paired Wilcoxon signed-rank tests were used with Benjamini-Hochberg correction. Bootstrap 95% confidence intervals are reported for paired differences and effect sizes.

Supplementary TableS6. Pairwise comparisons of readability outcomes

Metric	Comparison	N pairs	Mean difference	Mean difference 95% CI	Median difference	Median difference 95% CI	Rank-biserial correlation	Rank-biserial 95% CI	Unadjusted P	Adjusted P
ARI	ChatGPT vs Gemini	61	-3.37	-3.79--2.94	-3.58	-3.87--2.84	-0.994	-1.000--0.975	<0.001	<0.001
ARI	ChatGPT vs Copilot	61	-2.68	-3.15--2.17	-3.00	-3.37--2.04	-0.946	-0.994--0.869	<0.001	<0.001
ARI	ChatGPT vs DeepSeek	61	-4.04	-4.51--3.57	-4.12	-4.55--3.54	-1.000	-1.000--1.000	<0.001	<0.001
ARI	ChatGPT vs Doubao	61	-0.19	-0.63-0.26	-0.35	-0.65-0.18	-0.159	-0.460-0.123	0.281	0.281
ARI	Gemini vs Copilot	61	0.69	0.24-1.10	1.02	0.20-1.48	0.439	0.166-0.677	0.003	0.004

Metric	Comparison	N pairs	Mean difference	Mean difference 95% CI	Median difference	Median difference 95% CI	Rank-biserial correlation	Rank-biserial 95% CI	Unadjusted P	Adjusted P
ARI	Gemini vs DeepSeek	61	-0.68	-1.13--0.21	-0.44	-1.09-0.03	-0.362	-0.615--0.072	0.014	0.016
ARI	Gemini vs Doubao	61	3.17	2.73-3.60	3.41	2.64-3.90	0.976	0.915-1.000	<0.001	<0.001
ARI	Copilot vs DeepSeek	61	-1.36	-1.84--0.87	-1.26	-2.14--0.90	-0.674	-0.871--0.433	<0.001	<0.001
ARI	Copilot vs Doubao	61	2.49	2.01-2.94	2.64	1.62-3.18	0.929	0.835-0.989	<0.001	<0.001
ARI	DeepSeek vs Doubao	61	3.85	3.34-4.37	4.07	3.20-4.46	0.995	0.977-1.000	<0.001	<0.001
CL	ChatGPT vs Gemini	61	-2.76	-3.21--2.26	-3.00	-3.55--2.32	-0.933	-0.998--0.818	<0.001	<0.001
CL	ChatGPT vs Copilot	61	-3.02	-3.55--2.46	-3.29	-3.69--2.44	-0.944	-0.992--0.857	<0.001	<0.001
CL	ChatGPT vs DeepSeek	61	-2.82	-3.24--2.40	-2.93	-3.40--2.34	-0.978	-0.998--0.930	<0.001	<0.001
CL	ChatGPT vs Doubao	61	-0.78	-1.26--0.30	-0.79	-1.34--0.16	-0.433	-0.676--0.161	0.003	0.005
CL	Gemini vs Copilot	61	-0.26	-0.77-0.26	-0.17	-0.78-0.21	-0.151	-0.429-0.133	0.306	0.383
CL	Gemini vs DeepSeek	61	-0.06	-0.58-0.47	-0.05	-0.66-0.85	-0.030	-0.331-0.245	0.841	0.841
CL	Gemini vs Doubao	61	1.98	1.55-2.42	1.84	1.40-2.46	0.891	0.769-0.970	<0.001	<0.001
CL	Copilot vs DeepSeek	61	0.20	-0.23-0.62	-0.01	-0.40-0.81	0.113	-0.175-0.392	0.444	0.494
CL	Copilot vs Doubao	61	2.24	1.77-2.70	2.14	1.65-2.80	0.918	0.819-0.979	<0.001	<0.001
CL	DeepSeek vs Doubao	61	2.04	1.64-2.46	1.91	1.53-2.61	0.920	0.810-0.992	<0.001	<0.001

Metric	Comparison	N pairs	Mean difference	Mean difference 95% CI	Median difference	Median difference 95% CI	Rank-biserial correlation	Rank-biserial 95% CI	Unadjusted P	Adjusted P
FKGL	ChatGPT vs Gemini	61	-3.25	-3.73--2.79	-3.00	-3.84--2.64	-0.986	-1.000--0.953	<0.001	<0.001
FKGL	ChatGPT vs Copilot	61	-2.60	-2.96--2.22	-2.75	-3.10--2.44	-0.970	-1.000--0.915	<0.001	<0.001
FKGL	ChatGPT vs DeepSeek	61	-4.01	-4.51--3.52	-4.60	-5.15--3.98	-0.988	-1.000--0.959	<0.001	<0.001
FKGL	ChatGPT vs Doubao	61	-0.40	-0.84-0.04	-0.34	-0.93-0.20	-0.258	-0.525-0.024	0.081	0.081
FKGL	Gemini vs Copilot	61	0.66	0.21-1.08	0.42	0.13-1.28	0.388	0.119-0.635	0.009	0.011
FKGL	Gemini vs DeepSeek	61	-0.76	-1.37--0.15	-0.99	-1.85--0.47	-0.354	-0.600--0.066	0.016	0.018
FKGL	Gemini vs Doubao	61	2.85	2.36-3.34	3.13	2.83-3.33	0.948	0.870-0.991	<0.001	<0.001
FKGL	Copilot vs DeepSeek	61	-1.41	-1.87--0.91	-1.71	-2.35--1.01	-0.685	-0.859--0.473	<0.001	<0.001
FKGL	Copilot vs Doubao	61	2.19	1.76-2.62	2.26	1.81-2.80	0.926	0.830-0.992	<0.001	<0.001
FKGL	DeepSeek vs Doubao	61	3.60	3.09-4.11	3.65	3.10-4.09	0.989	0.959-1.000	<0.001	<0.001
GFI	ChatGPT vs Gemini	61	-2.92	-3.44--2.40	-2.56	-3.42--1.81	-0.973	-1.000--0.919	<0.001	<0.001
GFI	ChatGPT vs Copilot	61	-2.19	-2.71--1.67	-2.32	-2.76--1.98	-0.868	-0.957--0.741	<0.001	<0.001
GFI	ChatGPT vs DeepSeek	61	-3.10	-3.60--2.61	-2.97	-3.47--2.43	-0.990	-1.000--0.965	<0.001	<0.001
GFI	ChatGPT vs Doubao	61	-0.05	-0.54-0.45	-0.43	-0.90-0.65	-0.027	-0.330-0.250	0.858	0.858
GFI	Gemini vs Copilot	61	0.73	0.20-1.29	0.38	0.04-0.99	0.351	0.072-0.599	0.017	0.022

Metric	Comparison	N pairs	Mean difference	Mean difference 95% CI	Median difference	Median difference 95% CI	Rank-biserial correlation	Rank-biserial 95% CI	Unadjusted P	Adjusted P
GFI	Gemini vs DeepSeek	61	-0.18	-0.64-0.28	-0.23	-0.79-0.52	-0.108	-0.394-0.182	0.466	0.518
GFI	Gemini vs Doubao	61	2.87	2.32-3.41	2.89	2.15-3.08	0.969	0.892-1.000	<0.001	<0.001
GFI	Copilot vs DeepSeek	61	-0.91	-1.49--0.33	-0.71	-1.38-0.06	-0.401	-0.634--0.121	0.006	0.009
GFI	Copilot vs Doubao	61	2.14	1.63-2.62	2.10	1.69-2.60	0.857	0.725-0.956	<0.001	<0.001
GFI	DeepSeek vs Doubao	61	3.05	2.52-3.58	3.00	2.26-3.53	0.971	0.921-0.997	<0.001	<0.001
SMOG	ChatGPT vs Gemini	61	-2.69	-3.10--2.27	-2.48	-3.18--1.96	-0.992	-1.000--0.968	<0.001	<0.001
SMOG	ChatGPT vs Copilot	61	-2.08	-2.59--1.56	-1.98	-2.73--1.37	-0.850	-0.954--0.700	<0.001	<0.001
SMOG	ChatGPT vs DeepSeek	61	-2.94	-3.35--2.52	-2.85	-3.33--2.10	-0.995	-1.000--0.978	<0.001	<0.001
SMOG	ChatGPT vs Doubao	61	-0.79	-1.22--0.37	-0.57	-0.92--0.10	-0.434	-0.669--0.166	0.003	0.004
SMOG	Gemini vs Copilot	61	0.61	0.21-1.03	0.38	-0.12-1.11	0.357	0.091-0.598	0.015	0.017
SMOG	Gemini vs DeepSeek	61	-0.25	-0.60-0.10	0.06	-0.59-0.49	-0.151	-0.429-0.142	0.310	0.310
SMOG	Gemini vs Doubao	61	1.89	1.57-2.24	1.91	1.36-2.55	0.958	0.891-0.995	<0.001	<0.001
SMOG	Copilot vs DeepSeek	61	-0.86	-1.29--0.46	-0.83	-1.31--0.32	-0.507	-0.727--0.245	<0.001	<0.001
SMOG	Copilot vs Doubao	61	1.29	0.87-1.71	1.67	1.00-2.00	0.693	0.481-0.856	<0.001	<0.001
SMOG	DeepSeek vs Doubao	61	2.14	1.72-2.55	2.38	1.96-2.82	0.913	0.807-0.978	<0.001	<0.001

Metric	Comparison	N pairs	Mean difference	Mean difference 95% CI	Median difference	Median difference 95% CI	Rank-biserial correlation	Rank-biserial 95% CI	Unadjusted P	Adjusted P
FRES	ChatGPT vs Gemini	61	16.19	13.94-18.36	16.00	13.19-21.37	0.992	0.965-1.000	<0.001	<0.001
FRES	ChatGPT vs Copilot	61	12.04	9.65-14.47	12.21	9.93-16.09	0.914	0.816-0.977	<0.001	<0.001
FRES	ChatGPT vs DeepSeek	61	20.96	18.81-23.10	21.08	18.09-24.12	1.000	1.000-1.000	<0.001	<0.001
FRES	ChatGPT vs Doubao	61	4.26	2.10-6.41	4.40	1.70-7.35	0.512	0.259-0.740	<0.001	<0.001
FRES	Gemini vs Copilot	61	-4.15	-6.31--1.78	-4.43	-6.37--1.33	-0.466	-0.703--0.199	0.002	0.002
FRES	Gemini vs DeepSeek	61	4.77	2.85-6.79	5.31	1.63-7.38	0.601	0.363-0.804	<0.001	<0.001
FRES	Gemini vs Doubao	61	-11.93	-13.91--9.97	-10.29	-14.58--8.88	-0.975	-0.999--0.921	<0.001	<0.001
FRES	Copilot vs DeepSeek	61	8.92	6.80-11.08	7.72	4.93-11.34	0.891	0.773-0.976	<0.001	<0.001
FRES	Copilot vs Doubao	61	-7.78	-10.11--5.39	-9.04	-11.22--5.35	-0.735	-0.895--0.540	<0.001	<0.001
FRES	DeepSeek vs Doubao	61	-16.70	-18.47--14.93	-16.51	-19.71--14.45	-1.000	-1.000--1.000	<0.001	<0.001

Note: For FRES, higher scores indicate easier readability; for ARI, CL, FKGL, GFI, and SMOG, lower scores indicate easier readability.

Supplementary Table S7. Significant pairwise comparisons for non-binary outcomes

Metric	Comparison	N pairs	Mean difference	Mean difference 95% CI	Median difference	Median difference 95% CI	Rank-biserial correlation	Rank-biserial 95% CI	Unadjusted P	Adjusted P
Accuracy	ChatGPT vs Gemini	61	0.33	0.18-0.48	0.00	0.00-1.00	0.714	0.370-1.000	<0.001	<0.001
Accuracy	ChatGPT vs Copilot	61	0.75	0.59-0.90	1.00	1.00-1.00	0.959	0.864-1.000	<0.001	<0.001

Metric	Comparison	N pairs	Mean difference	Mean difference 95% CI	Median difference	Median difference 95% CI	Rank-biserial correlation	Rank-biserial 95% CI	Unadjusted P	Adjusted P
Accuracy	ChatGPT vs DeepSeek	61	0.92	0.77-1.07	1.00	1.00-1.00	0.966	0.889-1.000	<0.001	<0.001
Accuracy	ChatGPT vs Doubao	61	1.51	1.38-1.66	2.00	1.00-2.00	1.000	1.000-1.000	<0.001	<0.001
Accuracy	Gemini vs Copilot	61	0.43	0.25-0.61	0.00	0.00-1.00	0.804	0.490-1.000	<0.001	<0.001
Accuracy	Gemini vs DeepSeek	61	0.59	0.44-0.74	1.00	0.00-1.00	0.948	0.818-1.000	<0.001	<0.001
Accuracy	Gemini vs Doubao	61	1.18	1.05-1.31	1.00	1.00-1.00	1.000	1.000-1.000	<0.001	<0.001
Accuracy	Copilot vs Doubao	61	0.75	0.61-0.90	1.00	1.00-1.00	0.959	0.867-1.000	<0.001	<0.001
Accuracy	DeepSeek vs Doubao	61	0.59	0.46-0.70	1.00	0.00-1.00	1.000	1.000-1.000	<0.001	<0.001
Guideline Concordance	ChatGPT vs Gemini	61	3.49	2.30-4.61	4.00	3.00-5.00	0.756	0.542-0.935	<0.001	<0.001
Guideline Concordance	ChatGPT vs Copilot	61	7.67	6.79-8.49	8.00	7.00-8.00	0.993	0.971-1.000	<0.001	<0.001
Guideline Concordance	ChatGPT vs DeepSeek	61	8.80	7.90-9.66	9.00	9.00-10.00	0.992	0.968-1.000	<0.001	<0.001
Guideline Concordance	ChatGPT vs Doubao	61	14.10	12.90-15.16	15.00	14.00-16.00	0.998	0.987-1.000	<0.001	<0.001
Guideline Concordance	Gemini vs Copilot	61	4.18	3.30-5.16	4.00	3.00-5.00	0.966	0.894-1.000	<0.001	<0.001
Guideline Concordance	Gemini vs DeepSeek	61	5.31	4.39-6.41	5.00	4.00-6.00	0.974	0.913-1.000	<0.001	<0.001

Metric	Comparison	N pairs	Mean difference	Mean difference 95% CI	Median difference	Median difference 95% CI	Rank-biserial correlation	Rank-biserial 95% CI	Unadjusted P	Adjusted P
Guideline Concordance	Gemini vs Doubao	61	10.61	9.46-12.03	10.00	9.00-11.00	1.000	1.000-1.000	<0.001	<0.001
Guideline Concordance	Copilot vs DeepSeek	61	1.13	0.31-1.90	2.00	1.00-2.00	0.516	0.222-0.779	0.001	0.001
Guideline Concordance	Copilot vs Doubao	61	6.43	5.26-7.61	7.00	6.00-8.00	0.929	0.796-0.998	<0.001	<0.001
Guideline Concordance	DeepSeek vs Doubao	61	5.30	4.49-6.07	5.00	5.00-6.00	0.998	0.987-1.000	<0.001	<0.001
Empathy	ChatGPT vs Gemini	61	0.25	0.08-0.41	0.00	0.00-0.00	0.708	0.275-1.000	0.005	0.007
Empathy	ChatGPT vs Copilot	61	0.38	0.23-0.52	0.00	0.00-0.00	0.852	0.619-1.000	<0.001	<0.001
Empathy	ChatGPT vs DeepSeek	61	0.52	0.34-0.69	0.00	0.00-1.00	0.883	0.616-1.000	<0.001	<0.001
Empathy	ChatGPT vs Doubao	61	0.74	0.61-0.87	1.00	1.00-1.00	1.000	1.000-1.000	<0.001	<0.001
Empathy	Gemini vs DeepSeek	61	0.28	0.15-0.43	0.00	0.00-0.00	0.895	0.667-1.000	<0.001	<0.001
Empathy	Gemini vs Doubao	61	0.49	0.31-0.69	0.00	0.00-1.00	0.782	0.498-1.000	<0.001	<0.001
Empathy	Copilot vs Doubao	61	0.36	0.20-0.54	0.00	0.00-0.00	0.778	0.500-1.000	<0.001	<0.001
Empathy	DeepSeek vs Doubao	61	0.21	0.03-0.38	0.00	0.00-0.00	0.506	0.053-0.918	0.031	0.039
ARI	ChatGPT vs Gemini	61	-3.37	-3.79--2.94	-3.58	-3.87--2.84	-0.994	-1.000--0.975	<0.001	<0.001
ARI	ChatGPT vs	61	-2.68	-3.15--2.17	-3.00	-3.37--2.04	-0.946	-0.994--0.869	<0.001	<0.001

Metric	Comparison	N pairs	Mean difference	Mean difference 95% CI	Median difference	Median difference 95% CI	Rank-biserial correlation	Rank-biserial 95% CI	Unadjusted P	Adjusted P
	Copilot									
ARI	ChatGPT vs DeepSeek	61	-4.04	-4.51--3.57	-4.12	-4.55--3.54	-1.000	-1.000--1.000	<0.001	<0.001
ARI	Gemini vs Copilot	61	0.69	0.24-1.10	1.02	0.20-1.48	0.439	0.166-0.677	0.003	0.004
ARI	Gemini vs DeepSeek	61	-0.68	-1.13--0.21	-0.44	-1.09-0.03	-0.362	-0.615--0.072	0.014	0.016
ARI	Gemini vs Doubao	61	3.17	2.73-3.60	3.41	2.64-3.90	0.976	0.915-1.000	<0.001	<0.001
ARI	Copilot vs DeepSeek	61	-1.36	-1.84--0.87	-1.26	-2.14--0.90	-0.674	-0.871--0.433	<0.001	<0.001
ARI	Copilot vs Doubao	61	2.49	2.01-2.94	2.64	1.62-3.18	0.929	0.835-0.989	<0.001	<0.001
ARI	DeepSeek vs Doubao	61	3.85	3.34-4.37	4.07	3.20-4.46	0.995	0.977-1.000	<0.001	<0.001
CL	ChatGPT vs Gemini	61	-2.76	-3.21--2.26	-3.00	-3.55--2.32	-0.933	-0.998--0.818	<0.001	<0.001
CL	ChatGPT vs Copilot	61	-3.02	-3.55--2.46	-3.29	-3.69--2.44	-0.944	-0.992--0.857	<0.001	<0.001
CL	ChatGPT vs DeepSeek	61	-2.82	-3.24--2.40	-2.93	-3.40--2.34	-0.978	-0.998--0.930	<0.001	<0.001
CL	ChatGPT vs Doubao	61	-0.78	-1.26--0.30	-0.79	-1.34--0.16	-0.433	-0.676--0.161	0.003	0.005
CL	Gemini vs Doubao	61	1.98	1.55-2.42	1.84	1.40-2.46	0.891	0.769-0.970	<0.001	<0.001
CL	Copilot vs Doubao	61	2.24	1.77-2.70	2.14	1.65-2.80	0.918	0.819-0.979	<0.001	<0.001
CL	DeepSeek vs Doubao	61	2.04	1.64-2.46	1.91	1.53-2.61	0.920	0.810-0.992	<0.001	<0.001
FKGL	ChatGPT vs Gemini	61	-3.25	-3.73--2.79	-3.00	-3.84--2.64	-0.986	-1.000--0.953	<0.001	<0.001

Metric	Comparison	N pairs	Mean difference	Mean difference 95% CI	Median difference	Median difference 95% CI	Rank-biserial correlation	Rank-biserial 95% CI	Unadjusted P	Adjusted P
FKGL	ChatGPT vs Copilot	61	-2.60	-2.96--2.22	-2.75	-3.10--2.44	-0.970	-1.000--0.915	<0.001	<0.001
FKGL	ChatGPT vs DeepSeek	61	-4.01	-4.51--3.52	-4.60	-5.15--3.98	-0.988	-1.000--0.959	<0.001	<0.001
FKGL	Gemini vs Copilot	61	0.66	0.21-1.08	0.42	0.13-1.28	0.388	0.119-0.635	0.009	0.011
FKGL	Gemini vs DeepSeek	61	-0.76	-1.37--0.15	-0.99	-1.85--0.47	-0.354	-0.600--0.066	0.016	0.018
FKGL	Gemini vs Doubao	61	2.85	2.36-3.34	3.13	2.83-3.33	0.948	0.870-0.991	<0.001	<0.001
FKGL	Copilot vs DeepSeek	61	-1.41	-1.87--0.91	-1.71	-2.35--1.01	-0.685	-0.859--0.473	<0.001	<0.001
FKGL	Copilot vs Doubao	61	2.19	1.76-2.62	2.26	1.81-2.80	0.926	0.830-0.992	<0.001	<0.001
FKGL	DeepSeek vs Doubao	61	3.60	3.09-4.11	3.65	3.10-4.09	0.989	0.959-1.000	<0.001	<0.001
GFI	ChatGPT vs Gemini	61	-2.92	-3.44--2.40	-2.56	-3.42--1.81	-0.973	-1.000--0.919	<0.001	<0.001
GFI	ChatGPT vs Copilot	61	-2.19	-2.71--1.67	-2.32	-2.76--1.98	-0.868	-0.957--0.741	<0.001	<0.001
GFI	ChatGPT vs DeepSeek	61	-3.10	-3.60--2.61	-2.97	-3.47--2.43	-0.990	-1.000--0.965	<0.001	<0.001
GFI	Gemini vs Copilot	61	0.73	0.20-1.29	0.38	0.04-0.99	0.351	0.072-0.599	0.017	0.022
GFI	Gemini vs Doubao	61	2.87	2.32-3.41	2.89	2.15-3.08	0.969	0.892-1.000	<0.001	<0.001
GFI	Copilot vs DeepSeek	61	-0.91	-1.49--0.33	-0.71	-1.38-0.06	-0.401	-0.634--0.121	0.006	0.009
GFI	Copilot vs Doubao	61	2.14	1.63-2.62	2.10	1.69-2.60	0.857	0.725-0.956	<0.001	<0.001

Metric	Comparison	N pairs	Mean difference	Mean difference 95% CI	Median difference	Median difference 95% CI	Rank-biserial correlation	Rank-biserial 95% CI	Unadjusted P	Adjusted P
GFI	DeepSeek vs Doubao	61	3.05	2.52-3.58	3.00	2.26-3.53	0.971	0.921-0.997	<0.001	<0.001
SMOG	ChatGPT vs Gemini	61	-2.69	-3.10--2.27	-2.48	-3.18--1.96	-0.992	-1.000--0.968	<0.001	<0.001
SMOG	ChatGPT vs Copilot	61	-2.08	-2.59--1.56	-1.98	-2.73--1.37	-0.850	-0.954--0.700	<0.001	<0.001
SMOG	ChatGPT vs DeepSeek	61	-2.94	-3.35--2.52	-2.85	-3.33--2.10	-0.995	-1.000--0.978	<0.001	<0.001
SMOG	ChatGPT vs Doubao	61	-0.79	-1.22--0.37	-0.57	-0.92--0.10	-0.434	-0.669--0.166	0.003	0.004
SMOG	Gemini vs Copilot	61	0.61	0.21-1.03	0.38	-0.12-1.11	0.357	0.091-0.598	0.015	0.017
SMOG	Gemini vs Doubao	61	1.89	1.57-2.24	1.91	1.36-2.55	0.958	0.891-0.995	<0.001	<0.001
SMOG	Copilot vs DeepSeek	61	-0.86	-1.29--0.46	-0.83	-1.31--0.32	-0.507	-0.727--0.245	<0.001	<0.001
SMOG	Copilot vs Doubao	61	1.29	0.87-1.71	1.67	1.00-2.00	0.693	0.481-0.856	<0.001	<0.001
SMOG	DeepSeek vs Doubao	61	2.14	1.72-2.55	2.38	1.96-2.82	0.913	0.807-0.978	<0.001	<0.001
FRES	ChatGPT vs Gemini	61	16.19	13.94-18.36	16.00	13.19-21.37	0.992	0.965-1.000	<0.001	<0.001
FRES	ChatGPT vs Copilot	61	12.04	9.65-14.47	12.21	9.93-16.09	0.914	0.816-0.977	<0.001	<0.001
FRES	ChatGPT vs DeepSeek	61	20.96	18.81-23.10	21.08	18.09-24.12	1.000	1.000-1.000	<0.001	<0.001
FRES	ChatGPT vs Doubao	61	4.26	2.10-6.41	4.40	1.70-7.35	0.512	0.259-0.740	<0.001	<0.001
FRES	Gemini vs Copilot	61	-4.15	-6.31--1.78	-4.43	-6.37--1.33	-0.466	-0.703--0.199	0.002	0.002

Metric	Comparison	N pairs	Mean difference	Mean difference 95% CI	Median difference	Median difference 95% CI	Rank-biserial correlation	Rank-biserial 95% CI	Unadjusted P	Adjusted P
FRES	Gemini vs DeepSeek	61	4.77	2.85-6.79	5.31	1.63-7.38	0.601	0.363-0.804	<0.001	<0.001
FRES	Gemini vs Doubao	61	-11.93	-13.91--9.97	-10.29	-14.58--8.88	-0.975	-0.999--0.921	<0.001	<0.001
FRES	Copilot vs DeepSeek	61	8.92	6.80-11.08	7.72	4.93-11.34	0.891	0.773-0.976	<0.001	<0.001
FRES	Copilot vs Doubao	61	-7.78	-10.11--5.39	-9.04	-11.22--5.35	-0.735	-0.895--0.540	<0.001	<0.001
FRES	DeepSeek vs Doubao	61	-16.70	-18.47--14.93	-16.51	-19.71--14.45	-1.000	-1.000--1.000	<0.001	<0.001

Note: Only comparisons remaining significant after Benjamini-Hochberg correction are shown.

Supplementary Table S8. Significant pairwise comparisons of safety

Note
No significant pairwise safety difference after Benjamini-Hochberg correction.

Note: Only comparisons remaining significant after Benjamini-Hochberg correction are shown.
