# Supplementary material for: A comparative cross-sectional evaluation of generative AI chatbots for patient-oriented bacterial vaginosis health advice: safety, accuracy, guideline concordance, empathy, and readability
Source: Front Reprod Health. 2026 Sep 17;8:1967818. doi: 10.3389/frph.2026.1967818 (PMC13627438; doi:10.3389/frph.2026.1967818)
Supplement: Supplementary file 6 [file Datasheet7.pdf]

Supplementary Table S10. Sensitivity analysis comparing consensus ratings with median locked independent ratings

Outcome	Consensus-based primary analysis	Median locked-rating sensitivity analysis	Overall conclusion
Safety	Q = 2.596; P = 0.627	Q = 2.596; P = 0.627	Unchanged
Accuracy	χ^2 = 167.484; W = 0.686; P < 0.001	χ^2 = 169.383; W = 0.694; P < 0.001	Unchanged
Guideline concordance	χ^2 = 59.377; W = 0.243; P < 0.001	χ^2 = 59.377; W = 0.243; P < 0.001	Unchanged
Empathy	χ^2 = 78.600; W = 0.322; P < 0.001	χ^2 = 80.646; W = 0.331; P < 0.001	Unchanged

Chatbot	Question	Outcome	Consensus	Median locked rating
Gemini	60	Accuracy	3	4
Doubao	23	Empathy	5	4

Note: Consensus ratings were used in the prespecified primary analysis. The sensitivity analysis used the median of the five locked independent ratings for each response. Across the four clinician-rated outcomes, 1,218 of the 1,220 adjudicated response-level scores (99.8%) were identical to the corresponding median locked rating. The two discrepancies were the Accuracy rating for Gemini on Question 60 (consensus = 3; median locked rating = 4) and the Empathy rating for Doubao on Question 23 (consensus = 5; median locked rating = 4). The overall conclusions and the pattern of significance after Benjamini–Hochberg adjustment were unchanged.
